# Disseminated Coccidioidomycosis in an Adolescent With Crohn’s Disease

**DOI:** 10.7759/cureus.19980

**Published:** 2021-11-29

**Authors:** Rafael Gonzalez, Fouzia Naeem, Yoshihiro Ozaki, Vini Vijayan

**Affiliations:** 1 Pediatrics, Valley Children’s Healthcare, Madera, USA; 2 Pediatric Infectious Diseases, Valley Children’s Healthcare, Madera, USA

**Keywords:** san joaquin valley, immunocompromised host, biologic response modifiers, pneumonia, crohn’s disease

## Abstract

Coccidioidomycosis is a systemic mycosis caused by *Coccidioides immitis/posadasii. *This dimorphic fungus is endemic to the Southwestern United States, particularly in California and Arizona. Most infections are asymptomatic or mild, but around 5% of patients develop complicated pulmonary infection, and approximately 1% may progress to disseminated disease. We present the case of an adolescent male with Crohn’s disease who received the integrin inhibitor, vedolizumab, and developed disseminated coccidioidomycosis. This case underscores the importance of considering severe and/or disseminated coccidioidomycosis in immunosuppressed children. In our case, clinical suspicion and bronchoscopy helped confirm the diagnosis and facilitate appropriate evaluation and treatment.

## Introduction

Coccidioidomycosis is an endemic primary systemic mycosis caused by *Coccidioides immitis*. This dimorphic fungus is endemic to the Southwestern United States, particularly in California and Arizona and parts of Central and South America [1,2]. In the United States, the annual incidence of coccidioidomycosis is increasing, from a rate of 5.3 per 100,000 in 1998 to a rate of 42.6 per 100,000 in 2011 [3,4]. Pulmonary infection is caused by inhalation of spores (arthroconidia) of Coccidioides species after being released from the soil in endemic areas [4,5]. In normal hosts, most infections are asymptomatic or mild, but up to 5% of patients develop complicated pulmonary infection, and approximately 1% may progress to disseminated disease, which is associated with high morbidity and mortality [1,2,6,7].

We describe an adolescent Hispanic male with Crohn’s disease in remission who after receiving the integrin inhibitor, vedolizumab, presented with disseminated coccidioidomycosis. Vedolizumab is a biological response modifier known to have a gut-selective mechanism of action.

## Case presentation

An 18-year-old Hispanic male with Crohn’s disease was admitted to our institution with fever, cough, and progressive dyspnea. Four weeks prior to admission, the patient developed a productive cough associated with white sputum and dyspnea. He had no vomiting, diarrhea, or headache, but reported subjective fevers. The child was initially seen by his primary care physician and received azithromycin and intramuscular ceftriaxone without improvement. Past medical history was significant for Crohn’s disease diagnosed at the age of 3 years that was in remission on vedolizumab. Surgical history and family history were noncontributory. The patient lived with his mother in Central California. He denied animal exposure, sexual activity, smoking, vaping, or drug use. His immunizations were up to date.

Physical examination revealed an ill-appearing teenager in mild respiratory distress. His temperature was 38°C, blood pressure 122/64 mmHg, heart rate 102 beats/min, respiratory rate 28 breaths/min, and oxygen saturation rate 91% on room air. On auscultation, he had decreased air entry over the right lower lobe without rales or rhonchi. Abdominal examination was notable for diffuse tenderness with normoactive bowel sounds and hepatomegaly; however, negative for distension, guarding, and rebound. The remainder of his examination was normal.

Laboratory testing revealed white blood cell count of 22,000 cells/µL (78% neutrophils, 11% lymphocytes, 10% monocytes, 1% eosinophils), hemoglobin 10.2 g/dL, and platelets 409,000 cells/mm^3^. The C-reactive protein was 27.4 mg/dL and the erythrocyte sedimentation rate was 67 mm/hour. Blood cultures and viral respiratory panel were negative. Abdominal radiographs were negative for free air or obstruction. Chest radiography demonstrated right lower lobe pneumonia.

**Figure 1:**
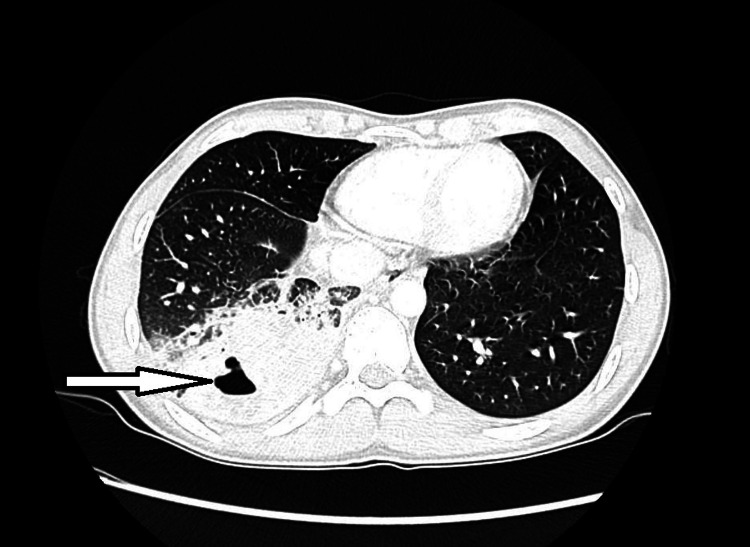
Axial chest CT in lung window demonstrating cavitary lesion within consolidated lung parenchyma. CT, computed tomography.

Upon admission, the child became febrile to 40.1°C, tachycardic, and hypotensive to 90/56 mmHg. He was started on cefepime, vancomycin, and liposomal amphotericin B (LAmB). LAmB was initiated due to concern for a fungal infection given child's geographical location. Computed tomography (CT) of the chest revealed right lower lobe pneumonia with central cavitation and right hilar and subcarinal adenopathy. Serology for cryptococcus, histoplasmosis, blastomycosis, and tuberculosis was negative. Urine legionella antigen was negative. He underwent a bronchoscopy and bronchoalveolar lavage (BAL). Cytology of BAL fluid showed the Gömöri methenamine silver stain positive spherules suspicious of *Coccidioides *sp. and culture grew *Coccidioides immitis* (Figure 1). His coccidioides complement fixation titer (CF) was 1:32.

**Figure 2:**
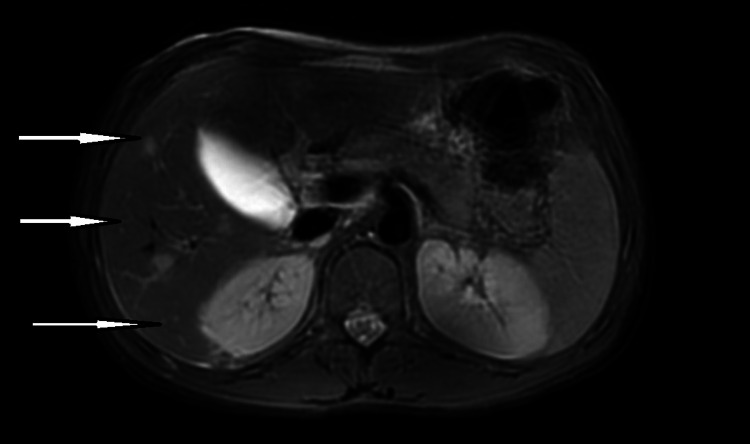
MRI abdomen post-contrast axial view demonstrating numerous scattered high-signal lesions throughout the liver. MRI, magnetic resonance imaging.

The patient was initially started on LAmB, and his vedolizumab infusion was discontinued. The *Coccidioides immiti*s isolate from his BAL showed a minimum inhibitory concentration (MIC) of 8 μg/mL to fluconazole. He was transitioned to fluconazole and discharged home after demonstrating an improvement in symptoms and a steady decline in coccidioides CF titer. However, 2 months later, his coccidioides CF titer increased to 1:512, concerning for disseminated disease. His nuclear medicine bone scan was negative for bony involvement, and cerebrospinal fluid analysis was unremarkable. Magnetic resonance imaging of the abdomen demonstrated the presence of multiple scattered lesions in the liver (Figure 2).

The patient was diagnosed with disseminated coccidioidomycosis (San Joaquin Valley fever) with pulmonary and hepatosplenic involvement. Relapse following successful treatment with fluconazole is a known challenge in the treatment of coccidioidomycosis and has been attributed to the inhibitory rather than fungicidal nature of fluconazole and may be related to the coccidioidal burden [8,9]. He was restarted on LAmB and underwent ultrasound-guided drainage of the hepatic lesion. *Coccidioides immitis* was isolated from the lesion and showed an MIC of 8 μg/mL to fluconazole. He completed 4 weeks of LAmB with significant clinical improvement and was discharged home on Itraconazole. The child has remained stable and shows no signs of disease progression or relapse at 1 year. His Crohn’s disease has remained in remission on enteral nutritional therapy alone.

## Discussion

Disseminated coccidioidomycosis generally occurs a few weeks to months after the initial infection, but reactivation of primary pulmonary coccidioidomycosis may occur at any time [5-7]. Patients with disseminated disease may have no radiographic evidence of previous pulmonary disease or respiratory illness preceding their presentation. The most frequent sites of extrapulmonary disease include the skin, lymph nodes, musculoskeletal, and central nervous system. Abdominal and pelvis coccidioidomycosis have also been described [7,10].

The risk of disseminated disease is higher in immunocompromised hosts. This includes children who receive immunosuppressive therapy, specifically agents that suppress cellular immunity such as corticosteroids, tumor necrosis factor-alpha (TNF-α) inhibitors, or chemotherapy [1,5-7]. The incidence of disseminated coccidioidomycosis is also higher among African Americans and Filipinos, Hispanics, Native Americans, and people living with diabetes mellitus or human immunodeficiency virus [1,5-7,11]. Our patient had several risk factors for disseminated disease, including Hispanic ethnicity, residence in an endemic area, and use of biological response modifier (BRM).

Over the past decade, the use of BRMs has become more widespread among children to induce and maintain remission of inflammatory bowel disease (IBD) [12,13]. A growing body of evidence highlights the association between the use of BRMs and increased risk of bacterial, viral, mycobacterial, and fungal infection. BRMs target and modify specific areas of the immune system and reduce the host immune response to inflammation [12-17]. A variety of BRMs exist for the treatment of IBD and include antagonists of TNF-α (infliximab, adalimumab, golimumab), integrin antagonists (vedolizumab), and other cytokines (ustekinumab) [12,13]. They target different proinflammatory proteins such as TNF-α, interleukin (IL)-12 and IL-23, and integrins. These proinflammatory proteins play a critical pathophysiologic role in initiating and sustaining inflammation in IBD [12,13].

Vedolizumab is an integrin antagonist. These transmembrane proteins influence cell signaling, and different integrin subunits are associated with white blood cell migration into the gastrointestinal tract. Vedolizumab specifically binds to the α4β7 integrin, which is expressed by gastrointestinal-homing T lymphocytes and blocks the binding of α4β7 integrin to mucosal addressin cell adhesion molecule-1, thereby inhibiting gastrointestinal inflammation [16,17].

Vedolizumab has a selective gastrointestinal mechanism of action without effects on circulating T-cells and, therefore, serious safety events and opportunistic infections are rare . When compared to TNF-α inhibitors, the risk of infections among patients who have received vedolizumab is low. However, it is important to note that the immunopathology of IBD is complex and there are many theories to suggest that Crohn’s disease may be a primary immunodeficiency state [18,19]. While the current understanding of this relationship is incomplete, individuals with IBD are predisposed to develop bacterial and viral infections and gastrointestinal infections and this risk is exacerbated with use of systemic corticosteroids and BRM. Our patient received vedolizumab due to failure to achieve suppression with conventional therapies for Crohn’s disease and presented with disseminated coccidioidomycosis. This is the first case to suggest an association between vedolizumab and disseminated coccidioidomycosis and further investigation is needed to elucidate a definitive cause and effect.

This patient completed 6 months of treatment with itraconazole but has not been restarted on a vedolizumab or another BRM as he has been in remission. Evidence-based data are needed to determine the factors that would make resuming a BRM safe after infection with coccidioidomycosis. Similarly, more research is needed to determine whether to screen children for endemic fungal infections before starting BRM and how to manage positive serologies in these children. Determining whether prophylaxis or suppressive therapy is warranted in such cases may help prevent morbidity and mortality associated with coccidioidomycosis. In a study performed by Wilson et al., it was determined that California spends an estimated $900 million a year in costs related to the care of individuals infected by coccidioidomycosis. Of this cost, $300 million is used to care for the estimated 200 people with disseminated coccidioidomycosis in the state [20]. As the population of endemic areas and the use of BRM increase in children [3], the diagnosis of management of coccidioidomycosis among children taking BRMs will remain a challenge for clinicians practicing in endemic regions.

## Conclusions

Our case underscores the importance of considering complicated pulmonary coccidioidomycosis and understanding the potential for severe and/or disseminated disease in immunosuppressed children, especially those on BRMs. In addition, this case highlights the need to maintain a high level of vigilance for disseminated disease among immunocompromised hosts with coccidioidomycosis to ensure that all potential sources of seeding are identified and managed accordingly. In our case, clinical suspicion and early bronchoscopy helped to confirm the diagnosis and facilitate appropriate evaluation and treatment.
